# 2411. Association between Causative Pathogen and Occurrence of Infection-Related Glomerulonephritis in Infective Endocarditis

**DOI:** 10.1093/ofid/ofad500.2031

**Published:** 2023-11-27

**Authors:** Nabin K Shrestha, Emese Kanyo, Georges Nakhoul, Leal Herlitz, Steven M Gordon

**Affiliations:** Cleveland Clinic Foundation, Cleveland, OH; Cleveland Clinic Lerner College of Medicine, Cleveland, OH; Cleveland Clinic, Cleveland, Ohio; Cleveland Clinic, Cleveland, Ohio; Cleveland Clinic Foundation, Cleveland, OH

## Abstract

**Background:**

Infection-related glomerulonephritis (IRGN) is an uncommon complication of infective endocarditis (IE). We have noticed that this complication occurs unusually frequently among patients with IE caused by *Bartonella* spp. The purpose of this study was to investigate the association between causative pathogen and the occurrence of IRGN in patients with IE.

**Methods:**

This was a retrospective case-control study. Episodes of pathologically-proven IE with a single causative pathogen, among patients admitted from July 1, 2007, through Jan 1, 2022 were identified from the Cleveland Clinic Infective Endocarditis Registry. Those with definite IRGN (proven by renal biopsy) or probable IRGN (hematuria, proteinuria, elevated creatinine, and depleted serum complement levels) were considered case subjects. Four control subjects were selected for each case subject from patients without definite, probable, or possible IRGN (hematuria, proteinuria, elevated creatinine, or hematuria, proteinuria, depleted serum complement levels), by propensity score matching on age, gender, admission year, and IE category (native or prosthetic), using the nearest neighbor method. The association between pathogen and occurrence of IRGN was examined using logistic regression.

**Results:**

Forty-eight case subjects (IE with IRGN) were matched to 192 control subjects (IE without IRGN). Of the 48 case subjects with IRGN, 17 (35%) had definite IRGN and 13 (27%) had prosthetic valve endocarditis. *Bartonella* was the causative pathogen in 9 (3.75%) subjects and of these, 8 had IRGN. There was a very strong association between *Bartonella* being the causative pathogen in IE and the occurrence of IRGN (OR 47.8, 95% C.I. 11.5 – 323.9, p-value < 0.001). A significant association with IRGN was not found for *Staphylococcus aureus*, viridans group streptococci, *Enterococcus*, coagulase-negative staphylococci, or fungi.

**Conclusion:**

*Bartonella* being the causative pathogen in IE is very strongly associated with the occurrence of IRGN. Acute glomerulonephritis in a person with clinical or echocardiographic features suspicious for IE, and negative blood cultures, should prompt specific testing for the presence of *Bartonella* infection by blood PCR or serology.

**Disclosures:**

**Leal Herlitz, MD**, Chemocentryx: Advisor/Consultant|Novartis: Advisor/Consultant

